# Saudi Medical Students' Perceptions and Attitudes of Integrating Generative Artificial Intelligence Integration in Medical Education: A Cross‐Sectional Study

**DOI:** 10.1002/hsr2.72904

**Published:** 2026-07-28

**Authors:** Fadi Aljamaan, Muhammad Faisal Mubarak, Ibraheem Altamimi, Alaa A. Alanteet, Mohammed A. Alsalman, Shereen A. Dasuqi, Rashid Alballaa, Mohammed I. Alarifi, Abdalrhman Al Saadon, Abdulrahman O. Alhaqbani, Abdulrahman A. Alhadlaq, Shirin H. Alokayli, Bader N. Alrasheed, Sarah I. Alkhalife, Kamran Sattar, Amr Jamal, Mona Soliman, Khaled Saad, Mohamad‐Hani Temsah

**Affiliations:** ^1^ College of Medicine King Saud University Riyadh Saudi Arabia; ^2^ Critical Care Department King Saud University Medical City, King Saud University Riyadh Saudi Arabia; ^3^ Department of Medicine, College of Medicine King Saud University Riyadh Saudi Arabia; ^4^ Department of Pharmacology and Toxicology, College of Pharmacy King Saud University Riyadh Saudi Arabia; ^5^ Department of Pharmacy, King Khalid University Hospital King Saud University Riyadh Saudi Arabia; ^6^ Medical Education Department, College of Medicine King Saud University Riyadh Saudi Arabia; ^7^ Department of Family Medicine King Saud University Medical City, King Saud University Riyadh Saudi Arabia; ^8^ AI Research Lab, College of Medicine King Saud University Riyadh Saudi Arabia; ^9^ Department of Pediatrics Assiut University Assiut Egypt; ^10^ Pediatric Department King Saud University Medical City, King Saud University Riyadh Saudi Arabia

**Keywords:** ChatGPT and digital learning, educational resources, generative artificial intelligence, LLM in higher education, Saudi Arabia AI adoption, undergraduate medical students

## Abstract

**Background and Aims:**

Generative Artificial Intelligence (GenAI) has catalyzed a transformation in medical education. Understanding learners' perceptions is essential to guide their responsible integration into curricula.

**Methods:**

A cross‐sectional survey was administered to 1039 undergraduate medical students across Saudi Arabia. A purpose‐developed, pilot‐tested instrument (Cronbach's *α* = 0.71 for dichotomous items) assessed students' familiarity with computational language models, perceptions of their educational utility, and attitudes toward technology‐enhanced pedagogical approaches. Descriptive statistics, Kolmogorov–Smirnov testing for normality, and multivariable binary logistic regression (two‐sided, *α* = 0.05) were performed using IBM SPSS Statistics v28.0.

**Results:**

Among 1039 participants (64.3% male; median age 22 years [IQR 20–24]), 57.2% (595/1039) reported familiarity with computational language models in medical education, and 70.1% (728/1039; 95% CI: 67.2–72.9) supported curricular integration. A strong majority (86.4%; 898/1039; 95% CI: 84.2–88.4) anticipated impact on the future of medical education. While 73.4% (763/1039; 95% CI: 70.6–76.0) perceived benefit for basic science education, only 41.6% (432/1039; 95% CI: 38.6–44.6) recognized utility in clinical skills training. Only 29.8% (310/1039; 95% CI: 27.0–32.7) considered these tools superior to human instruction. Key concerns included distrust in output reliability (52.6%; 547/1039; 95% CI: 49.5–55.7) and awareness of reference fabrication (64.0%; 665/1039; 95% CI: 61.0–66.9).

**Conclusion:**

Saudi medical students express strong interest in GenAI—particularly for basic sciences and simulation—but perceive it as complementary rather than superior to human instruction. Findings reflect learner perceptions, not measured educational effectiveness. Implementation should prioritize reliability, ethical use, and preservation of humanistic competencies.

**Abbreviations**
GenAI: generative artificial intelligence
LLMs: large language models

## 1 Introduction

During the last century, the landscape of medical care underwent a transformative evolution, from the development of mega‐sized literature databases that spread scholars' achievements across the globe, to various technological and digital achievements that boosted medical care and outcomes in the last 50 years [1]. Medical education, which is the foundation source for producing capable healthcare professionals, has been affected by this revolution [2]. Educational domains such as simulation, clinical skills, and patient care training have been the major fields of evolution in this regard.

The recent COVID‐19 pandemic, with its lockdowns and social distancing measures, has significantly accelerated the adoption of telemedicine, virtual training, and online education [3]. The pandemic paved the road for rapid integration and advancements of artificial intelligence (AI) technologies as part of digital health, which has been adopted and integrated broadly after the pandemic [4]. OpenAI's Chat Generative Pre‐Trained Transformer (ChatGPT) is one of the pioneer advanced large language models (LLMs) that have been launched and have taken multiple evolutionary steps recently [5]. In medical education, this evolution is more than a technological leap; it signifies a paradigm shift in how medical students and tutors access, interact with, and process medical and non‐medical information [6].

Notably, AI platforms and similar LLMs are opening new dimensions for integrating AI into healthcare. LLM technology shows promise in supporting learners and tutors, assisting with learner assessments, reviewing educational curricula, and ensuring alignment with educational objectives [7, 8]. LLMs have been shown to rival conventional information sources, such as search engines, in terms of their output, summarization capabilities, organization, and relevance to search queries. However, a user preference emerges: traditional search engines are typically preferred for direct, fact‐based inquiries, whereas LLMs are more frequently chosen for tasks that require nuanced comprehension and sophisticated language processing [9, 10]. While preliminary studies have highlighted LLMs' potential to provide medical students with information across various specialties, with varying reliability, there remains a critical need to ensure their standardization, credibility, integrity, and ethical use [11, 12, 13].

Usage of AI chatbots was associated with criticism in terms of their output's credibility and information sources' validity [14]. A study examining the implementation of ChatGPT in medical education revealed its valuable and efficient role in information gathering and summarization. However, concerns were noted about its content's critical depth, ambiguous sourcing, and reliability, highlighting a need for a methodology to critically appraise its output and deal with ChatGPT in a balanced, careful strategy [15]. Another valued potential of LLMs in medical education is procedural and simulation education. Multiple studies that assessed digital feedback on procedural skills training have shown promising learning outcomes [16, 17], while other studies also showed some negative outcomes that directed attention to protocolizing and evaluating such computer‐assessed skills learning in order to improve outcomes and avoid any unintended disadvantages [18]. AI technology has also been implemented in learning objectives assessment with variable success [19, 20, 21]. It is imperative to adopt AI technology in teaching methods and enhance their outcomes, in addition to constructing ethics of conduct for its use among medical students, ensuring they are well‐equipped for this rapidly evolving digital era [22, 23]. This paper assessed medical students' perspectives on artificial intelligence as a medical education tool, enabling medical educators and academics to navigate the challenges and opportunities presented in this rapidly evolving era of LLMs.

While the technical potential and educational applications of LLMs have been widely discussed, there is a need for a comprehensive domain‐ and concept‐based assessment of medical students' perceptions and attitudes toward integrating these technologies into current medical curricula, taking into consideration their perceptions of AI's impact on patient care and healthcare system outcomes. Research on students' views, particularly in the early stages of generative AI adoption, is relatively limited, as it has not delved deeply into multiple domains of medical education, including the assessment process. Understanding these perceptions is critical for the effective and responsible integration of AI in medical education.

This study aims to fill this gap by assessing undergraduate medical students' perceptions and attitudes toward the integration of AI technologies, specifically generative AI tools, within medical education in Saudi Arabia. The insights gained will help guide educators and policymakers in understanding how students view AI and its potential impact across various educational domains. This research aims to contribute to the global conversation on AI in education and provide valuable insights into its role in shaping the future of medical training. While prior investigations have examined learners' attitudes toward digital educational tools in high‐income settings, this study offers original contextual insights by capturing perceptions from a large, geographically diverse cohort of Saudi undergraduate medical trainees during the early adoption phase of advanced natural language processing technologies. The investigation further contributes by examining perceptions across multiple educational domains (basic sciences, clinical teaching, simulation, assessment) within a single, unified survey instrument.

## 2 Methods

### 2.1 Study Design

Cross‐sectional survey‐based exploratory and item‐based rather than a unified psychometric scale study targeting undergraduate medical students. The questionnaire used in this study was specifically developed by the research team to assess medical students' perceptions and attitudes towards generative AI in medical education.

The survey development process involved a comprehensive literature review and adoption from previous studies that targeted the same or similar objectives. The work was followed by four focus group discussions (FGDs) to refine and finalize the survey, based on the expected domains of Generative Artificial Intelligence integration and employment in medical education, as well as anticipated challenges and impacts on medical education, patient care, and the healthcare sector in general. The final survey was reviewed by a multidisciplinary team comprising a pediatric intensivist professor (MHT), an adult intensivist (FA), a medical educationist (MS), a biostatistician (MA), and a group of undergraduate medical students at different levels (IA, MAA, AOA, AAA, SHA, BNA, SIA). This diverse team of experts empowered the survey's robustness by focusing on its content, validity, and relevance. The refined survey was then pilot‐tested with 30 medical students at various academic levels to ensure its content validity, clarity, and suitability. Feedback from this pilot study led to further refinement, enhancing the survey's overall structure and reliability.

The survey was structured into three parts. The first part gathered information about participants' gender, medical education study level, computer skills expertise, familiarity with the role of artificial intelligence in medical education, and their accessibility to chatbots. The second part assessed participants' perceptions of the impact of artificial intelligence on the future of medical education, their opinions on incorporating this technology into their curriculum, the future of medical practice, and their perceptions of it as a medical information source. Also, we assessed participants' opinions about the role of generative AI in medical education, probing into its potential impact across various domains of medical education, that is, basic sciences, clinical sciences, simulation, skill‐based teaching and academic assessment and evaluation process, comparing AI to human tutor through all these different domains, additionally we assessed participants' about their awareness of AI Chatbots hallucinations/fabrications and whether they perceive this as an obstacle for their integration in medical education methodology. We also assessed participants' perceptions of AI relative to traditional medical teaching methods and the impact of AI chatbots on their academic achievement. The questionnaire consisted of multiple‐choice questions, encompassing a range of response types, and trichotomous questions (yes/no) with a neutral choice. This design allowed us to capture a comprehensive spectrum of responses. The survey was distributed to a diverse group of medical students to cover the wide range of academic levels across universities with colleges of medicine in KSA.

### 2.2 Participant Recruitment and Sampling Methodology

To effectively gather data from a diverse range of medical students across Saudi Arabia, the research team adopted a digital‐centric approach for distributing the survey. The survey was hosted on SurveyMonkey due to its proven efficiency in electronic dissemination and data export for statistical analysis. The study was conducted over a 4‐week period from October 1 to October 30, 2023. We used convenience sampling techniques, utilizing social media platforms widely used by the medical student community, such as X and WhatsApp, along with email invitations and personal contacts of the research team. The study aimed to ensure wide geographic coverage and inclusiveness. The inclusion criteria consisted of all undergraduate medical students from any medical school in Saudi Arabia. This method not only facilitated the recruitment of a broad spectrum of participants from various regions of Saudi Arabia but also aligned with the high engagement of medical students on these platforms, meeting the study's inclusion criteria.

### 2.3 Sample Size

The required sample size was calculated using the Raosoft Sample Size Calculator [24]. The calculated sample size required was 386 medical students, assuming an estimated proportion of medical students using AI chatbots of 50%, a margin of error of 5%, a 95% confidence level, and a study power of 80%. To accommodate potential incomplete responses and non‐responses, the sample size was increased by 20%, resulting in a minimum of 463 medical students.

### 2.4 Ethical Considerations

This study was conducted in accordance with the ethical principles outlined in the Declaration of Helsinki. The study received ethical approval from the Institutional Review Board (IRB) at King Saud University, Riyadh, Saudi Arabia (Approval # E‐23‐7847). The purpose of the study was clearly outlined on the first page of the electronic survey, where informed consent was also obtained. For any queries, participants were given the option to contact the principal investigator via email. Participation in this study was entirely voluntary, and no financial or other incentives were provided to participants. Participants were informed that they could withdraw from the study at any time without facing any penalty or consequence. To maintain confidentiality, no personal identifiers were collected from the respondents.

### 2.5 Statistical Analysis

Statistical analysis was conducted in accordance with the SAMPL guidelines and recommendations for reporting statistics in clinical research. Categorical variables were summarized as frequencies and percentages with exact numerators and denominators. Continuous variables were assessed for normality using the Kolmogorov–Smirnov test and visual inspection of histograms; non‐normally distributed data are presented as medians with interquartile ranges (IQRs). Internal consistency for dichotomous items was evaluated using the Kuder–Richardson formula 20 (KR‐20 = 0.71). Multivariable binary logistic regression models were constructed to examine associations between participant characteristics and key perceptual outcomes. Results are reported as adjusted odds ratios (aOR) with 95% confidence intervals (CIs) and two‐sided *p*‐values. All analyses were prespecified except subgroup explorations noted in tables. Variance inflation factors were examined for all multivariable regression models; values < 2.5 indicated acceptable multicollinearity. Given the conceptual overlap among perceptual variables (e.g., beliefs regarding reliability, educational utility, and ethical acceptability), analytical interpretations are framed cautiously, recognizing that these constructs represent related but distinct attitudinal dimensions. Missing data were handled via listwise deletion; sensitivity analyses were not performed due to low missingness (< 2%). SPSS IBM statistical software version 28 for Windows (IBM Corp., Armonk, NY) was used for statistical data analysis. The alpha significance level was set at 0.050.

## 3 Results

**Figure 1:**
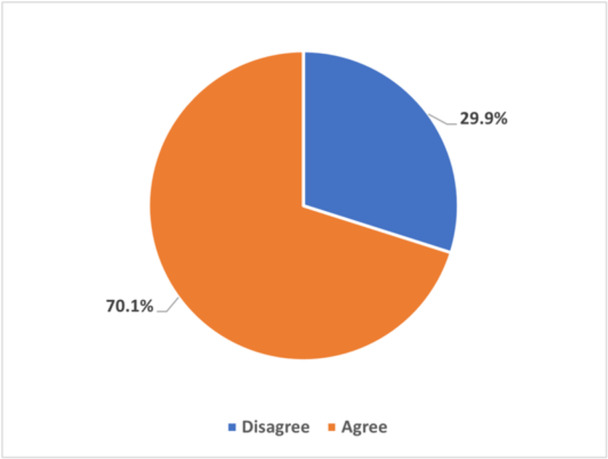
Participants' opinions regarding the incorporation of generative artificial intelligence (GenAI) technologies into the teaching methodology of their medical school (*N* = 1039). The bar chart displays the proportion of undergraduate medical students who agreed (70.1%; *n* = 728) versus disagreed (29.9%; *n* = 311).

A total of 1039 medical students participated in our study, with a predominant male representation of 64.3% (668/1039). The participants were split between junior medical students in basic science or non‐clinical years (40.7%) and senior students in clinical years (47.3%), with 12% in their internship year. The vast majority (76.1%) expressed very good to excellent computer skills. As shown in Tables 1, 57.2% (595/1039; 95% CI: 54.2–60.3) of participants were familiar with AI chatbots, compared to 42.8% who were not. The vast majority (75.5%) admitted being able to access AI chatbots (Table 1). As shown in Tables 1, 57.3% were familiar with the AI role in medical education, while 86.4% believed AI would impact the future of medical education, and 70.1% agreed with incorporating AI technology into their medical school curriculum (Figure 1).

**Table 1:** Descriptive analysis of medical students' sociodemographic and academic characteristics (*N* = 1039).

	Frequency	Percentage
Sex		
Female	371	35.7
Male	668	64.3
Study Level		
Junior students	423	40.7
Senior students	491	47.3
Intern	125	12
Location		
Central region Riyadh	858	82.6
Other provinces	181	17.4
Participants' computer skills		
Poor	29	2.8
Fair	220	21.2
Very good	516	49.7
Excellent	274	26.4
Accessibility to AI Chatbots		
No	255	24.5
Yes	784	75.5
Participants' familiarity with the AI role in medical education		
No	444	42.7
Yes	595	57.3
Do you think AI Chatbots would impact medical education future?		
No	141	13.6
Yes	898	86.4

**Figure 2:**
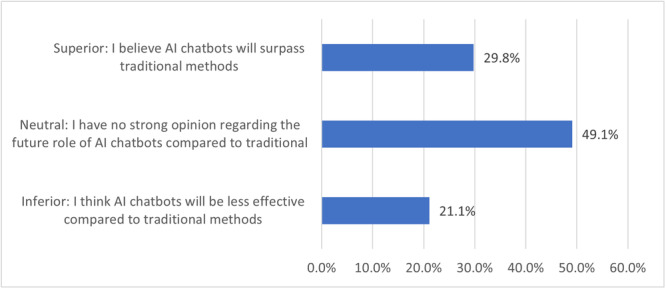
Participants' perceptions comparing the anticipated effectiveness of generative artificial intelligence (GenAI) technologies versus traditional teaching methods in medical education (*N* = 1039). The chart illustrates the distribution of responses across three categories: (1) Inferior—students who believe AI chatbots will be less effective compared to traditional methods (21.1%; *n* = 219); (2) Neutral—students with no strong opinion regarding the future role of AI chatbots compared to traditional teaching (49.1%; *n* = 510); and (3) Superior—students who believe AI chatbots will surpass traditional methods (29.8%; *n* = 310).

Participants' perception of AI technology impact in medical education is shown in Table 2, where 73.4% believed AI chatbots will have an impact on the basic sciences portion of medical education, followed by simulation‐based teaching, 58.1%, clinical sciences, 54.9%, and only 41.6% with an impact on clinical skills teaching (Table 2). The vast majority (80%) did not think ‘AI‐harnessed medical teaching technology’ would be more informative than ‘traditional human tutor’ for clinical sciences, and 62.4% did not think so for basic sciences, as shown in Table 2. On the other hand, 51.7% believed that “AI‐assisted academic assessment” would be fairer and more objective compared to “human tutoring.” The participants were split equally regarding their perceptions of the ethicality of using AI chatbots in their assignments. While 60.7% of the participants believed that AI technology would improve their academic achievements, 9.3% believed there was no effect, and the rest were undecided (Table 2). Only 29.8% of participants believed that AI technology would be superior to the current traditional teaching methodology in medical schools, while 21.1% believed the opposite, and the remainder were undecided (Figure 2).

**Table 2:** Participants' perception of AI implementation in medical education (*N* = 1039).

	Frequency	Percentage
Which of the following is a potential impact of AI in medical education		
Clinical sciences (e.g., Internal Medicine, Obstetrics and Gynecology)	570	54.9
Basic sciences (e.g., Physiology, Anatomy)	763	73.4
Simulation	604	58.1
Clinical Skills (e.g., Physical examination, surgical techniques)	432	41.6
Other (Assignments and Homework)	36	3.5
Do you think AI as a tutor would be more efficient and informative than a human tutor for basic science teaching?		
No	648	62.4
Yes	391	37.6
Do you think AI as a tutor would be more efficient and informative than a human tutor for clinical teaching, especially skills?		
No	831	80
Yes	208	20
Do you think AI AI‐based assessment versus human tutor would be fairer and more objective?		
No	502	48.3
Yes	537	51.7
Do you think medical students’ usage of AI Chatbots in assignment completion is ethical?		
No	519	50
Yes	520	50
Participants’ perception of AI Chatbots’ contribution to medical students’ academic achievement		
No	97	9.3
Neutral	311	29.9
Yes	631	60.7
Are AI Chatbots reliable sources of information for medical students?		
No	547	52.6
Yes	492	47.4
Are you aware of references to fabrication/hallucination encountered with AI Chatbots?		
No	619	59.6
Yes	420	40.4
Are AI Chatbots' references to confusion/hallucination an obstacle to their use in medical education?		
No	374	36
Yes	665	64

Only 47.4% believed AI chatbots as reliable sources of information for medical students. In the same context, 40.4% were aware of AI chatbot references' fabrication/hallucination, with 64% of participants perceived this fallacy to pose an obstacle to incorporating them into medical education (Table 2).

We also assessed participants' perceptions of the impact of AI on the future of medical practice, as shown in Table 3, and found that 71.1% believed physicians' access to AI would affect their future competencies and efficiencies. While 44.7% believed it might lead to the replacement of some medical specialties in the future, 33.8% did not share this view, and 21.6% were undecided in that regard. On the other hand, 61.1% believed AI technology would improve medical practice and patient care outcomes.

**Table 3:** Participants' perception about AI and the future of medical practice (*N* = 1039).

	Frequency	Percentage
Do you think physician access to AI Chatbots in the future would affect their competencies and efficiency?		
No	300	28.9
Yes	739	71.1
Do you think AI technology will improve medical practice?		
No	115	11.1
Neutral	300	28.9
Yes	624	60.1
Do you think AI technology will improve patient care?		
No	131	12.6
Neutral	284	27.3
Yes	624	60.1
Do you think AI technology might replace some of the medical specialties in the future?		
No	351	33.8
Neutral	224	21.6
Yes	464	44.7

Table 4 presents a multivariate binary logistic regression analysis of participants' characteristics associated with their perception of AI technology's positive impact on the future of medical education. Males were significantly less inclined to believe in the positive impact of AI technology on the future of medical education compared to females (39% less, *p* = 0.028). Medical students who agreed with the superiority of AI over traditional tutors in terms of clinical teaching efficiency and informativity were significantly less inclined to believe in its future positive impact on medical education (50.4% less, *p* = 0.006). However, students who encouraged the incorporation of AI technology in medical schools' teaching methods, and those who believed AI chatbots are reliable sources of medical information, were significantly more inclined to believe in their positive impact on the future of medical education (3.83 times more, *p* < 0.001, 1.926 times more, *p* = 0.003), respectively.

**Table 4:** Multivariable logistic binary regression analysis of participants' belief in the impact of AI technology on medical education future.

	Multivariate adjusted odds Ratio	OR 95% CI		*p* value
		Lower	Upper	
Sex	0.610	0.393	0.948	0.028
Age (years)	0.978	0.895	1.069	0.627
AI is more efficient and informative for clinical teaching	0.496	0.300	0.819	0.006
Encourages AI incorporation in medical teaching methodology	3.583	2.401	5.347	< 0.001
AI Chatbots are a reliable source of information for medical students	1.926	1.244	2.980	0.003
AI would improve medical students’ academic achievement	0.765	0.610	0.959	0.020
AI will improve medical practice	0.597	0.466	0.766	< 0.001
AI will improve patient care	0.764	0.602	0.970	0.027
Constant	45.762			0.000

*Note:* DV: Participants' belief in the impact of AI technology on the future of medical education.

Abbreviations: CI, confidence interval; OR, odds ratio.

On the other hand, students who believed AI chatbots could improve medical students' academic achievement were significantly less inclined to believe in AI technology's positive impact on the future of medical education (23.5% less likely, *p* = 0.020). Similarly, those who believed that AI technology would improve medical practice and patient care were significantly less optimistic about its future in medical education (40.3% less, *p* < 0.001; 23.5% less, *p* = 0.027). Table 5 shows the multivariable binary logistic regression analysis for the odds of participants' characteristics associated with their agreement to incorporate AI technology in medical education methodology. Male medical students were significantly less likely than females to agree with incorporating AI technology into medical education methodology (32.4% fewer agreeing, *p* = 0.022). Senior medical students (22 years or older) were significantly more likely to agree with incorporating AI technology into medical education (1.57 times more likely, *p* = 0.003) than their peers. Medical students who had access to AI chatbots were significantly more likely to agree to incorporate AI technology into medical schools' teaching methodologies (59.5%, *p* = 0.005). Medical students who believed in the usefulness of AI chatbots in medical simulation‐based teaching and their superiority to a human tutor for teaching basic science were significantly more likely to incorporate them into medical schools’ teaching methodologies (1.439 times more, *p* = 0.015, and 2.117 times more, *p* < 0.001, respectively). Medical students who believed that AI‐assisted academic assessment would be fairer and more objective than human evaluators were significantly more likely to agree with incorporating AI technology into medical schools' teaching methodologies (1.667 times more, *p* = 0.001). The medical students who believed that using AI chatbots in assignment completion is ethical were significantly more in agreement with incorporating this technology into the teaching methodology (1.593 times more, *p* = 0.002). On the other hand, those who believed that AI chatbot usage would enhance their academic achievement were significantly less likely to agree to incorporate AI technology into their educational methodology (28.3% less likely, *p* < 0.001). Medical students who perceived that AI technology would improve patient outcomes were significantly less likely to agree to incorporate it into their medical schools’ teaching methodology (24.4% less likely, *p* = 0.001).

**Table 5:** Multivariable logistic binary regression analysis of participants' agreement with AI incorporation in medical school education methodology.

	Multivariate adjusted Odds Ratio	OR 95% CI		*p* value
		Lower	Upper	
Sex	0.676	0.484	0.946	0.022
Age group	1.572	1.163	2.123	0.003
University location	1.419	0.911	2.210	0.122
AI Chatbots ease of access	1.595	1.152	2.209	0.005
AI has a potential impact on simulation‐based teaching	1.439	1.073	1.929	0.015
AI is more efficient and informative for basic sciences	2.117	1.531	2.927	< 0.001
AI‐based academic assessment would be fairer and more objective	1.677	1.247	2.256	0.001
Medical students' usage of AI Chatbots in assignments completion is ethical	1.593	1.185	2.142	0.002
AI would improve medical students' academic achievement	0.717	0.609	0.844	< 0.001
AI will improve patient care	0.756	0.640	0.893	0.001
Constant	1.844			0.070

*Note:* DV: participants' agreement with AI incorporation in medical school education methodology.

Abbreviations: CI, confidence interval; OR, odds ratio.

Table 6 sheds light on the characteristics of participating medical students associated with their belief in the reliability of AI chatbots as a medical information source. Male medical students had significantly lower beliefs than female medical students (31.6% lower, *p* = 0.008). Those who strongly believed in AI's role in simulation teaching had significantly less confidence in its information source reliability (24.1% less, *p* = 0.048). While those who believed AI would be more efficient than human tutors in basic science, clinical skills teaching, and potentially a fairer evaluator had significantly higher trust in AI's medical information reliability (66.5%, *p* = 0.001; 82.4%, *p* = 0.002; 36.2%, *p* = 0.029), respectively. Medical students who believed that using AI chatbots in assignment completion was ethical had significantly higher beliefs in the reliability of their medical information (63.2%, *p* < 0.001). On the other hand, those who expected AI to have a positive impact on the future of medical education had significantly less belief in the reliability of AI chatbots' medical information (21.4% less, *p* = 0.003).

**Table 6:** Multivariable logistic binary regression analysis of medical students' odds of believing in the reliability of AI chatbots as a source of medical information.

	Multivariate adjusted Odds ratio	OR 95% CI		*p* value
		Lower	Upper	
Sex	0.684	0.518	0.904	0.008
Age(years)	0.979	0.922	1.039	0.477
AI usefulness in medical simulation (Clinical scenarios)	0.759	0.577	0.998	0.048
AI would be more efficient than a human tutor for basic science teaching.	1.665	1.242	2.233	0.001
AI would be more efficient than a human tutor for clinical skills teaching	1.824	1.257	2.647	0.002
AI‐based academic assessment would be fairer and more objective.	1.362	1.033	1.796	0.029
Medical students' usage of AI Chatbots in assignments completion is ethical.	1.632	1.246	2.137	< 0.001
AI Chatbots would impact medical education future.	2.237	1.450	3.453	< 0.001
Perceives AI has a superior impact in medical education compared to traditional methodology	0.800	0.682	0.939	0.006
AI Chatbots reference that hallucination is an obstacle to their application in medical education.	0.662	0.501	0.874	0.004
AI will improve medical practice	0.786	0.670	0.921	0.003
Constant	1.419			0.636

*Note:* DV: Participants' belief in AI chatbots as a reliable source for medical education.

Abbreviations: CI, confidence interval; OR, odds ratio.

Medical students' belief that AI might have an impact on the future of medical education correlated significantly with their belief in its information reliability (22.3 times more, *p* < 0.001), but those who believed this impact is due to its superiority compared to traditional medical education methods had less trust in its medical information reliability (20% times less, *p* = 0.006). However, medical students' belief that AI chatbots' hallucinations would be an obstacle to their incorporation in medical education was associated with a lower belief in the reliability of their medical information (33.2% less, *p* = 0.004).

The characteristics of participating medical students associated with their perception of AI hallucination as an obstacle to its implementation in medical education are presented in Table 7. Those who believed AI would enhance medical practice and those who believed they are reliable sources for medical information were significantly less likely to perceive their hallucination as a barrier for use in medical education (16.9% times less, *p* = 0.019, 30.9% times less, *p* = 0.009), respectively. Medical students who were aware of the AI chatbots' hallucination phenomenon were significantly more likely to predict (3.50 times more, *p* < 0.001) that their hallucination would be a barrier to their use in medical education. Medical students who agreed that the AI role in the future of medical teaching would surpass traditional medical methods were significantly more inclined to perceive AI chatbot hallucination as a barrier to its use in medical teaching (1.307 times more, *p* = 0.001). Additionally, medical students who agreed that physicians' access to AI chatbots in the future may affect their efficiency and competence were significantly more likely to perceive the hallucination of chatbots as a barrier to their use in medical education (1.357 times more, *p* = 0.039). The odds that participating in medical students perceive future physicians' access to AI chatbots as affecting their competency and efficiency are shown in Table 8.

**Table 7:** Multivariable logistic binary regression analysis of participants' odds of perceiving AI chatbots' hallucination as an obstacle in their implementation in medical education.

	Multivariate adjusted Odds Ratio	Or 95% C.I.		
		Lower	Upper	*p*‐value
Sex	0.716	0.539	0.952	0.021
Age (years)	0.994	0.937	1.055	0.850
Participants’ belief in AI potential to enhance medical practice	0.831	0.712	0.970	0.019
Participants’ aware of the references to hallucination encountered with AI Chatbot usage.	3.503	2.608	4.705	< 0.001
AI Chatbots are a reliable source of information for medical students.	0.691	0.524	0.911	0.009
Perception AI chatbots’ role in the future of medical education compared to traditional methods	1.307	1.112	1.536	0.001
Physician’ access to AI Chatbots in the future would affect their competencies and efficiency.	1.357	1.015	1.813	0.039
Constant	1.178			0.820

*Note:* DV: AI Chatbots references confusion/hallucination as an obstacle to its use in medical education.

Abbreviations: CI, confidence interval; OR, odds ratio.

**Table 8:** Multivariable logistic binary regression analysis of participants' belief in the effect of Physician access to AI Chatbots on their future competencies and efficiency.

	Multivariate adjusted Odds ratio	OR 95% CI		
		Lower	Upper	*p*‐value
Sex	1.064	0.795	1.423	0.679
Age (years)	0.992	0.933	1.054	0.786
Participants' level in medical school Senior	0.673	0.550	0.822	< 0.001
AI Chatbots' reference to hallucination is an obstacle to their use in medical education.	1.452	1.094	1.928	0.010
Participants' belief in AI's potential to improve patient care	0.788	0.674	0.921	0.003
AI‐based academic assessment versus human tutor would be fairer and more objective.	1.418	1.071	1.878	0.015
Participants' expectations of AI's potential to replace some of the medical specialties in the future	0.744	0.623	0.889	0.001
Constant	9.539			0.002

*Note:* DV: Physician access to AI Chatbots in the future would affect their competencies and efficiency.

Abbreviations: CI, confidence interval; OR, odds ratio.

Senior medical students had lower beliefs that access to an AI chatbot might affect their competencies and efficiency (32.7% lower, *p* < 0.001). At the same time, students who had a belief in AI's potential to improve patient care, and those who expected that it might replace some medical specialties in the future, also had lower belief in the effect of physician access to AI chatbots on their future competencies (21.2% less, *p *= 0.003, 25.6% less, *p*‐value 0.001).

Students who believed the AI chatbot hallucination phenomenon might be an obstacle to its future use in medical education had higher beliefs in AI chatbots' effects on physicians’ future competencies and efficiency (45.2% more, *p* = 0.01).

## 4 Discussion

Our study is powered by the inclusion of 1039 medical students. Most of them (75.5%) admitted being able to access AI chatbots, yet they were less familiar with areas where it could be applied in medical education (57%). Our substantial number of participants’ familiarity with AI chatbots correlates with similar literature findings ranging from 50% to 85% [25, 26, 27, 28]. Our finding of low curiosity and interest at the current stage in Generative AI technology in medical education points to medical students’ hesitance and reluctance toward its role in medical education. At the same time, the majority (86.4%) of participants believed in AI's inevitable future impact on medical education, as supported by similar surveys [29]. In comparison to our studied cohort, Jordanian medical students, who are very close geographically, sociologically, and technologically to our population, had a lower expectation (67%) of AI's potential to revolutionize medical education [27].

Female students in our study, compared to males, held stronger beliefs about the AI's impact on the future of medical education, were more encouraged to incorporate it into the medical education curriculum, and trusted AI chatbots as a reliable source of medical information. The literature showed inconsistent findings regarding sex differences in terms of medical and allied healthcare professional students’ optimism for the AI future and its impact on medical education [30, 31, 32, 33, 34].

Our observed high participants’ expectations of AI's future impact on medical education might be driven by their perception of its multidimensional tutorship efficiency, including basic sciences, simulation teaching to some extent, and its potential role to improve students’ academic achievement, which mirrors similar survey studies regarding medical students’ optimism in terms of AI technology application in medical education and assignment preparation [27]. We did not measure directly patient care outcomes in our study, but interestingly, those who perceived a potential positive AI impact on clinical practice and patient care outcomes did not support incorporating AI technology in medical education. This domain‐specific expectations of AI incorporation in the medical field might be explained by their perception of AI technology's role as more befitting administrative, logistical, and organizational roles rather than educational applications [35], or because they perceived academics and students’ skills are still lacking and precluding AI application into medical education, as observed similarly in a previous survey [28].

Notably, 71.1% of participants believed that physicians’ access to AI chatbots would affect their future clinical and career competency, but did not perceive that this access would improve patients’ care and medical practice, probably because they perceive medical practice as complex and requires a human physician approach not yet achieved by clinical AI [28]. Chen's systematic review has yielded similar findings, with a consensus on the need for collaboration between clinical AI and human physicians, despite mixed opinions about its future as a surrogate physician [36]. Therefore, medical students and physicians are encouraged to develop and optimize their AI technology competency to keep pace with future healthcare systems that will inevitably incorporate clinical Generative AI technology across various sectors [37]. 70% of participants agreed that incorporating AI technology into their medical school curriculum would be beneficial. However, when questioned about AI technology's possible superiority compared to traditional teaching methodologies, only 29.8% expected its superiority, and 21.1% expected it would be inferior; the majority were undecided. Medical students demonstrated inconsistent or reluctant views on the impact of AI on different medical education domains, especially when compared to traditional human tutors. They were optimistic about its educational role for basic sciences rather than simulation and clinical medicine teaching. This differential optimism is probably explained by the students’ perception of AI's role at the current stage as an information source that summarizes and organizes the educational material in a concise format, rather than a tutoring or mentoring tool, achieved by human tutoring skills of professionalism and critical thinking skills that cannot be replaced so far by artificial intelligence technology. Current literature supports concerns about AI applications in medical education, including the potential for inadvertent or intentional overreliance on AI by medical students, the evanescence of critical thinking skills, and the reliability of LLM output [14]. For the sake of fairness, AI has been widely accepted for its tutoring capabilities, especially in surgical skills training [38].

One possible challenge of incorporating AI into medical education is the lack of reliable information. Only 47.4% of our participants believed AI chatbots would act as a reliable source of information and predicted a high impact on medical education in the future, yet they also perceived them as lacking. Another challenge is the widely reported phenomenon in the medical literature of information and reference fabrication/hallucination encountered while using AI applications [39, 40, 41], as 64% of survey takers believed it might be an obstacle for AI chatbots’ incorporation into medical education. Those concerned about AI chatbots’ hallucinations had negative expectations about their impact on physicians’ future competencies and perceived this phenomenon as an obstacle to AI incorporation in medical education.

Senior medical students (those in their 3rd to 5th years of medical school) had lower expectations that AI technology would affect their future competencies and efficiency than basic science students or junior students (those in their 1st and 2nd years of medical school). This may be related to the advanced level of senior medical students, especially given their exposure to clinical medicine practice. 44.7% of our participants expected AI technology to replace some medical specialties in the future, although they did not perceive clinical AI as having an adverse effect on their future clinical competency or proficiency. This observation might be explained by perceiving AI technology to be inferior to human competency due to humans’ cumulative training, acquired clinical critical thinking skills, and humanistic approach to patients. Our findings align with the reviewed contextual literature, which suggests that AI will only act to complement and reinforce human physician skills, rather than replace them [42, 43].

Students who perceived AI as fairer and more objective in academic assessment compared to human tutors were strongly encouraged to incorporate it into medical education methodology and had higher trust in it as a source of medical information. Furthermore, the same group of students had positive perceptions of its impact on medical education and healthcare practice. This has been observed in the literature and credited as an advantageous characteristic for AI implementation in the education field [44]. This extreme enthusiasm may stem from biased, unrealistic expectations of the applied AI role in medical education and practice, lacking a substantive basis. Medical students may perceive AI‐administered evaluations as free from the personal biases that human evaluators might encounter [44]. However, similarly, AI technology might introduce another element of bias, which is dependent on algorithms and protocols programmed to feed AI platforms during prompting and evaluation processes. Therefore, the implementation of AI in medical education assessment should be protocolized, objectively and skeptically, and carefully employed to achieve a comprehensive, unbiased assessment process.

Students who believed in AI's superior role in simulation and basic science teaching, compared to clinical teaching, had a strong inclination to incorporate AI into medical education and perceived a positive impact on the future of medical education. Participants who believed in AI's superiority in tutoring clinical and basic sciences also strongly believed in the reliability of AI chatbots’ information. These findings reveal domain‐positive expectations among survey participants: one group strongly believes in the application of AI for information retrieval, while the other perceives the usefulness of AI in virtual and robotic applications, as well as in information retrieval and diagnostic tools.

Participants’ optimism regarding the future educational role of computational language models coexisted with reservations about their applicability to direct patient care. Importantly, this study assessed learner perceptions only; no objective measures of clinical outcomes, patient safety, or healthcare system performance were collected. Statements regarding patient care reflect participant beliefs, not empirical evidence of effectiveness. A plausible explanation to this contradiction could be explained by participants perception of AI domain role in medical education in comparison to medical practices and its outcomes, and the participants’ immature understanding of the clinical care process and healthcare systems as various AI applications in the medical field have proven to improve overall patient care especially when it comes to medical decision making in complex cases, inventing personalized therapies for patient with frequent hospital admissions, aiding surgeons during operation, and fine‐tuning infection control within a healthcare system [45].

## 5 Limitations

Our survey inherently has potential limitations. Our participant's geographic representation has majorly covered the Riyadh region which included about 7 medical schools with much less representation of the rest of KSA, this potentially can bias medical students perceptions against AI adoption in medical education, especially those in rural areas might not be exposed to same digital and technological advances as those in the capital region (Riyadh), therefore the generalizability of our results is limited to medical students who are digitally active and competent in those skills. Another potential limitation of our survey was that it was launched early in the adoption of generative AI applications in medical education, which may have limited participants’ insights into its long‐term impact. Their modest familiarity with AI applications at the time could have influenced their perceptions. Additionally, we did not explore students’ understanding of deep learning or the evolution of LLMs, nor did we assess educators’ perspectives, which are crucial for integrating AI.

The survey instrument, while pilot‐tested and content‐validated, remains exploratory in psychometric structure. Domain‐level reliability and factor structure were not formally evaluated; thus, analytical interpretations should be tempered accordingly. Additionally, multivariable regression models included conceptually related perceptual variables as both predictors and outcomes; variance inflation factors were examined (< 2.5 for all models), but residual confounding by unmeasured attitudinal constructs cannot be excluded. Findings represent cross‐sectional perceptions and do not assess longitudinal educational outcomes or comparative instructional effectiveness.

Furthermore, our recruitment strategy, primarily utilizing social media platforms (X and WhatsApp) and personal contacts, introduced specific limitations. While these channels allowed for a wide geographic reach, they may have led to a selection bias, potentially overrepresenting medical students who are more engaged with digital platforms. Despite the widespread use of social media among Saudi medical students [46]. This method does not guarantee a completely random sample of the entire population. Consequently, we were unable to calculate a traditional response rate, as the total number of individuals exposed to the survey could not be precisely determined. This limitation limits the generalizability of our findings, suggesting that the results may be more representative of medical students active on these digital platforms than of the entire Saudi medical student population.

Future research should investigate faculty viewpoints, assess AI's long‐term impact on medical training, and explore the effectiveness of advanced AI models like GPT‐o1 or DeepSeek and other domain‐specific LLMs in clinical education and decision‐making [37].

## 6 Conclusions

AI applications have promising roles in medical education, such as providing medical information resources, enriching simulation, and enhancing robotic medical education, including surgical skills, as well as optimizing basic sciences teaching in anatomy and physiology, for example. These potentials should be highlighted to medical students and academics. Probably, AI is still lacking clinical education skills due to multiple challenges in comparison to human tutors, such as professionalism, critical thinking, and empathetic medical practice. The overzealous use of AI by medical students raises serious concerns regarding potential misuse and overreliance. AI excels in its organizing and summarizing skills, which should be highlighted. At the same time, attention should be drawn to the information sources and reliability, especially for junior users, such as medical students. Therefore, there is an urgent and ongoing need to address the accuracy of AI applications’ outputs in the medical field, to mature and optimize their implementation in the medical education process.

## Author Contributions

**Fadi Aljamaan:** conceptualization, investigation, funding acquisition, writing – original draft, methodology. **Muhammad Faisal Mubarak:** conceptualization, investigation, funding acquisition, writing – original draft. **Ibraheem Altamimi:** investigation, funding acquisition, writing – original draft. **Alaa A. Alanteet:** formal analysis, data curation. **Mohammed A. Alsalman:** data curation, formal analysis, writing – review and editing. **Shereen A. Dasuqi:** data curation, formal analysis, writing – review and editing. **Rashid Alballaa:** data curation, formal analysis, visualization. **Mohammed I. Alarifi:** formal analysis. **Abdalrhman Al Saadon:** data curation, formal analysis, visualization, validation, methodology. **Abdulrahman O. Alhaqbani:** methodology, data curation. **Abdulrahman A. Alhadlaq:** formal analysis. **Shirin H. Alokayli:** methodology, visualization, writing – review and editing, data curation, project administration. **Bader N. Alrasheed:** methodology, validation, visualization, project administration, formal analysis. **Sarah I. Alkhalife:** methodology, project administration, data curation. **Kamran Sattar:** data curation, supervision, resources, software. **Amr Jamal:** software, visualization, resources. **Mona Soliman:** software. **Khaled Saad:** writing – review and editing, visualization, validation, formal analysis, supervision. **Mohamad‐Hani Temsah:** conceptualization, investigation, funding acquisition, writing – original draft, methodology; validation, visualization, software, formal analysis, project administration, resources, supervision.

## Funding

Ongoing Research Funding Program (ORF‐2026‐2027), King Saud University, Riyadh, Saudi Arabia.

## Ethics Statement

The study was approved by the Institutional Review Board at King Saud University, Riyadh, Saudi Arabia (IRB Approval # E‐23‐7847). The study was conducted in accordance with the principles outlined in the Declaration of Helsinki.

## Conflicts of Interest

The authors declare no conflicts of interest.

## Transparency Statement

The lead author, Mohamad‐Hani Temsah, affirms that this manuscript is an honest, accurate, and transparent account of the study being reported; that no important aspects of the study have been omitted; and that any discrepancies from the study as planned (and, if relevant, registered) have been explained.

## Data Availability

The data that support the findings of this study are available from the corresponding author upon reasonable request. The corresponding author, Khaled Saad, had full access to all data in this study and takes full responsibility for the integrity and accuracy of the data analysis.
